# Soluble PD-L1 Expression After Intravenous Treatment of Cancer Patients With Selenite in Phase I Clinical Trial

**DOI:** 10.3389/fonc.2022.906134

**Published:** 2022-06-02

**Authors:** Ali Razaghi, Ladan Mansouri, Ola Brodin, Mikael Björnstedt, Joachim Lundahl

**Affiliations:** ^1^ Division of Pathology, Department of Laboratory Medicine, Karolinska Institutet, Karolinska University Hospital Huddinge, Stockholm, Sweden; ^2^ Karolinska Institutet, Clinical Science and Education, Södersjukhuset, Stockholm, Sweden; ^3^ Theme Cancer, Karolinska University Hospital, Solna, Sweden

**Keywords:** cancer, clinical trial, PD-L1, selenium, selenite

## Abstract

A high expression level of programmed death-ligand 1 (PD-L1) is observed in different types of cancers (particularly lung cancer). Soluble (s)PD-L1 may be used as a prognostic marker and a target for anti-cancer immunity, as well as, predicting gene therapy or systemic immunotherapy in blocking the PD-1 and PD-L1 checkpoint. Studies that evaluate the effects of the immune regulator selenium on PD-L1 expression show ambiguous results. Thus, we aimed to analyze sPD-L1 levels in samples from patients who underwent different dosages of selenite treatment in phase I clinical trial. We hypothesized that selenite modulates the sPD-L1 levels in the plasma as a consequence of the suggested mode of action of selenotherapy in cancer patients. In conclusion, our results support the view that selenotherapy does not substantially affect the PD-1/PD-L1 axis judged by sPD-L1 analysis. Furthermore, no significant correlation was observed between the survival and sPD-L1 expression nor sPD-L1 changes. However, due to a dynamic individual sPD-L1 profile and a high variation in survival, we suggest that further studies are needed to identify whether individual patients can be benefited from combinational seleno- and anti-PD-L1 therapy.

## Introduction

A high expression level of programmed death-ligand 1 (PD-L1, CD274), an essential immune checkpoint protein, is observed in different types of cancers, particularly lung cancer. The expression is regulated by multiple signaling pathways such as NFκB, MAPK, and mTOR (1). The PD-L1 binds to programmed death 1 (PD-1) on T-lymphocytes which results in tumor immunosuppression *via* reducing T cell activity and proliferation (1). To date, several PD-L1 or PD-1 monoclonal antibodies (e.g., durvalumab and pembrolizumab) have been approved by the U.S. Food and Drug Administration (FDA) for the treatment of multiple cancer types like melanoma, non-small-cell lung cancer, and gastric cancer exhibiting meaningful clinical benefits with response rates of ~40% (1).

Selenium, an essential trace element, regulates immune functions through the redox activity of selenoproteins, which protects immune cells from oxidative stress. Thus far, many studies have shown that selenium intake may reduce the risk of cancer incidence by reversing the immunosuppression in the tumor microenvironment towards antitumor immunity by activation of immune cells (e.g. CD8+ T-lymphocytes) (2).

The studies that evaluate the effects of selenium on PD-L1 expression disclose ambiguous results. For example, it was suggested that methylseleninic acid enhanced T cell-mediated killing of ovarian cancer cells *via* PD-L1 inhibition in an *in vitro* study (3). In addition, selenium nano-particles (composed of 1_8 μM sodium selenite) downregulated the expression of PD-1 and PD-L1 on cytokine-induced killer cells and hepatocellular carcinoma cells *in vivo* (4). In contrast, the expression of PD-L1 was not changed following 3 months of supplementation with selenium (200 µg.day^-1^) in a randomized clinical trial of lymphoma patients (5).

To further gain insight into the clinical potential of selenotherapy, in 2015, we published a first-in-man systematic phase I clinical trial (the SECAR study) in patients with cancer (IV to end-stage) and we could demonstrate that sodium selenite is safe and tolerable if given below the maximum tolerated dose (10.2 mg.m^2^ body surface area) (6).

Soluble (s)PD-L1 is mainly produced through proteolytic cleavage of membrane-bound PD-L1. Parallel elevation of both sPD-1 and sPD-L1 suggests their regulatory properties balance each other’s effects as in the case of membrane-bound up-regulation (7). For example, patients with hepatocellular carcinoma had a 2 fold-increase in sPD-1 and sPD-L1 following sorafenib treatment (8). In addition, therapeutic anti-PD-L1 monoclonal antibodies can be deactivated by high levels of sPD-L1 in plasma before reaching the tumor site. Thus, evaluation of sPD-L1 levels may predict the effectiveness of the anti-PD-L1 therapy (9). Overall, sPD-L1 plays a role in cancer prognosis and anti-cancer immunity, as well as predicting gene therapy or systemic immunotherapy in blocking the PD-1 and PD-L1 checkpoint interaction (7).

Given a potentially important relationship between the PD-L1 axis and selenium, we analyzed sPD-L1 levels in samples from patients who underwent different dosages of selenite treatment in the SECAR study (6). We hypothesized that selenite modulates the sPD-L1 levels in the plasma as one mechanism of the suggested mode of action of selenotherapy in cancer patients.

## Methods

### Study Population and Treatment

The phase I clinical trial was an open-label dose-escalation study of intravenous administered sodium selenite (Intro-Selen i.v., Pharma Nord ApS, Denmark) as a single agent.

Due to the limited volume of samples, out of a total of 35 individuals involved in the SECAR clinical trial, we could use samples from 24 patients in this study who were diagnosed with varied types of cancer, comprising of non-small cell lung cancer (n=15), colon cancer (n= 4), small cell lung cancer (n=1), rectal cancer (n=1), tongue cancer (n=1), ethmoidal cancer (n=1), testicular cancer (n=1) and malignant mesothelioma (n=1). The median age of the cohort is 62 years old. All patients had advanced carcinomas and had been treated with established chemotherapy for respective carcinoma.

The participants were divided into different subgroups based on the dosage of selenite (1, 1.5, 2, 3, 4.5, 6.8, 10.2, 12.8, and 15.3 mg.m^2^). The analysis was conducted in samples collected at three-time points; day 1 before the first treatment (BT), day 5-12 treatment period (TP), and at the end of treatment (EoT) (between 1 and 9 weeks after the last selenite treatment). Blood samples were collected ~5 min before respective treatments (**Table 1**). The plasma fractions were obtained by centrifugation and stored at -80°C until further analyses. A detailed study design and patient information including cancer types, stage of cancer, and chemotherapy regimen are presented in the previously published article (6). Patients were not treated with PD-1/PD-L1 inhibitors.

**Table 1:** Dose exposure and treatment design. **OPD,** once per day.

Dosage (mg.m^2^)	No. of Patients	Treatment Schedule
1	3	OPD; 5 day/week; 4 weeks
1.5	2	OPD; 5 day/week; 4 weeks
2	2	OPD; 5 day/week; 4 weeks
3	3	OPD; 5 day/week; 2 weeks
4.5	3	OPD; 5 day/week; 2 weeks
6.8	2	OPD; 5 day/week; 2 weeks
10.2	3	OPD; 5 day/week; 2 weeks
12.8	2	OPD; 5 day/week; 2 weeks
15.3	4	OPD; 5 day/week; 2 weeks

### ELISA

The plasma levels of soluble PD-L1 (sPD-L1) were assessed at 600 nm wavelength (Labsystems, Multiskan Ascent V1.25) using the human PD-L1 ELISA Kit (catalog #ab214565; Abcam) following the manufacturer’s instruction.

### Statistics

#### Analysis of PD-L1 Expression

Data analysis was performed using one-way ANOVA (paired) PrismGraphPad 8.3.1 assuming Gaussian distribution. The alpha value was set to (α =0.05). If one-way ANOVA was significant, the *post hoc* Tukey test with a 90% confidence interval was performed comparing multiple treatments.

#### Correlation Analysis

Correlation analysis between PD-L1 expression BT, TP, EoT, and delta value (EoT-TP) versus survival was performed using PrismGraphPad 8.3.1 assuming Gaussian distribution. Both Pearson correlation coefficient and nonparametric Spearman correlation were computed. The P-value (two-tailed) was analyzed with a 95% confidence interval.

## Results

**Figure 1:**
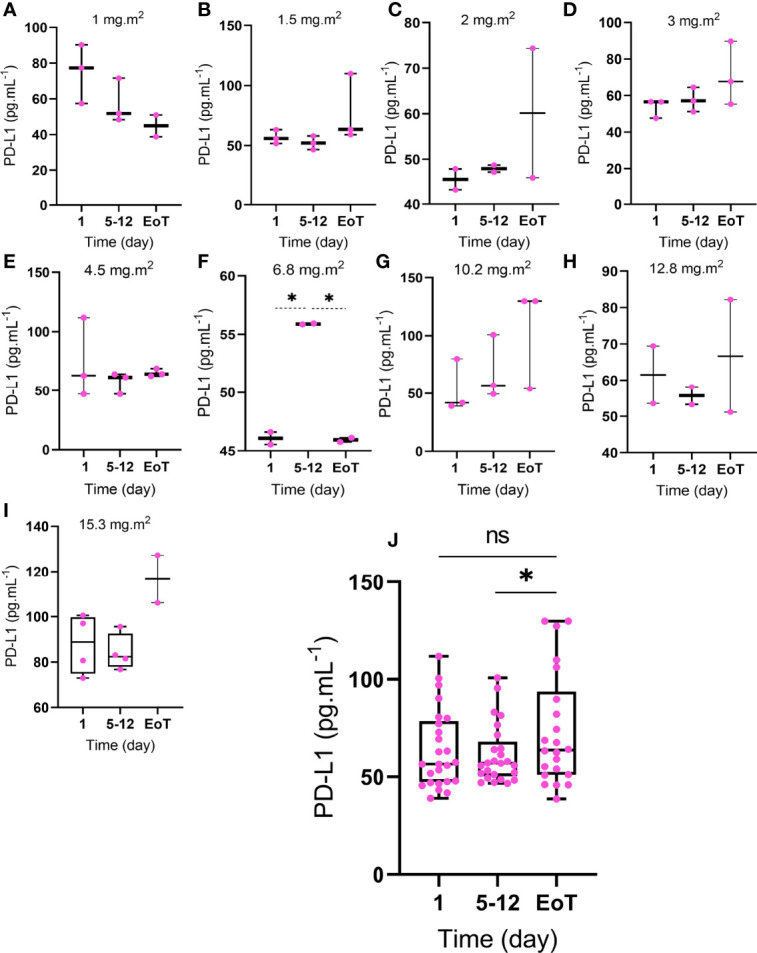
Expression of PD-L1 in cancer patients following treatment with selenite. The participants are divided into different subgroups based on the dosage of selenite **(A)** 1, **(B)** 1.5, **(C)** 2, **(D)** 3, **(E)** 4.5, **(F)** 6.8, **(G)** 10.2, **(H)** 12.8, and **(I)** 15.3 mg.m^2^. The analysis was conducted in three-time points; BT: day 1 before treatment, TP: day 5-12 treatment period), and EoT: end of treatment. The combined data is shown (**Figure 1J**). Statistical significance (p ≤ 0.05) is marked by an asterisk (*). ns: not significant. (n=3, MEAN ± SD).

When all the data was combined regardless of dosage, one-way ANOVA showed that EoT was significantly higher than TP. However, the EoT level did not significantly differ from BT(**Figure 1J**). The only significant differences between BT and TP as well as EoT were observed in 2 patients treated with a dosage of 6.8 mg.m^2^ (**Figure 1F**).

Only one of the patients (KL004) showed striking clinical benefits including shrinkage of tumor (squamous cell lung carcinoma) 4 months after receiving selenite therapy (1 mg. m^2^) and 10 months later disappearance of tumor according to PET-CT scan. After the complete response, she lived 7 years without recurrence and any more cancer treatment. This patient was the only patient whose sPD-L1 expression decreased more than 50% comparing BT to EoT. However, no statistically significant difference was observed in the sPD-L1 level before and at the end of treatment in the entire cohort i.e. sPD-L1 levels did not reflect clinical outcome and thus may not be used as a predictive marker.

Furthermore, no significant correlations were found between survival versus BT, TP, EoT, and delta value (EoT-TP). A poor prognosis was found also in our patients since the median survival for all patients was 6.6 months (if the cured patients were excluded).

## Discussion

In this study, we demonstrate that sPD-L1 levels, as a marker for the activity in the PD-1/PD-L1, axis display a dynamic profile during selenotherapy without any clear association with clinical data. We could not identify any association on an individual level between clinical outcome, the dose of selenium given, and changes in sPD-L1 levels (**Figures 1A–E, G–I**), which makes sPD-L1 levels unsuitable to be used as a biomarker for treatment efficacy.

This study indicates an increase in sPD-L1 levels at the end of treatment compared to samples collected during the treatment period (**Figure 1J**). This finding is of interest since a post-therapeutic increase in sPD-1 plasma level is correlated with improved survival for various types of cancers while elevated plasma levels of sPD-L1 have been correlated with poor prognosis in cancer (7). Also, high sPD-L1 seems to be associated with clinical characteristics of NSCLC (10). Furthermore, a phase III trial of combination chemotherapy (rituximab, cyclophosphamide, anthracycline, vincristine, and prednisone) in patients with large B-cell lymphoma showed that 30% of the patient cohort had high sPD-L1 plasma levels and sPD-L1 plasma concentration ≥1.52 ng.mL^-1^ was a predictor of poor prognosis (11).

Our data indicate that selenite treatment in cancer patients does not noticeably affect the level of PD-L1 expression. However, we notice a marked dynamic and individual change in sPD-L1 levels before, during, and after selenite treatment as well as a high variation in survival. In general, our result is contrary to preclinical (*in vitro* and *in vivo*) studies which have suggested PD-L1 expression to be inhibited following selenium treatment (3, 4). Furthermore, when considering sPD-L1 as a biomarker or drug target, the structural heterogeneity of sPD-L1 proteins, as well as the associated functional/cellular plurality, should be taken into account because sPD-L1, as an integral part of the dynamic PD-1/PD-L1 signaling pathway, is known to be essential for both immune-tolerance or immune-escape (12). On the other hand, our data is in accordance with the results of a randomized clinical trial showing that selenium supplementation in lymphoma patients does not change the level of PD-L1 protein expression (5). The difference between preclinical versus clinical results can be interpreted based on the fact that overall concordance between preclinical toxicology and clinical safety data is poor because infections or other indirect outcomes of immunomodulation, as well as cytokine-associated phenomena, are not modulated in preclinical studies (13).

## Limitations

The most prominent limitation of this study is the small number of patients. Tissue samples are also required for further research to elucidate the significance of PD-L1 expression. Thus, more confirmatory studies, validation, and new studies comparing sPD-L1 *vs* membrane PD-L1 are needed to define the prognostic and predictive value of these potential biomarkers following selenotherapy.

In some patients, especially those receiving selenite treatment above the maximum tolerated dose (15.3 mg.m^2^) the treatment was withdrawn in advance due to toxicity.

## Conclusion

**Figure 2:**
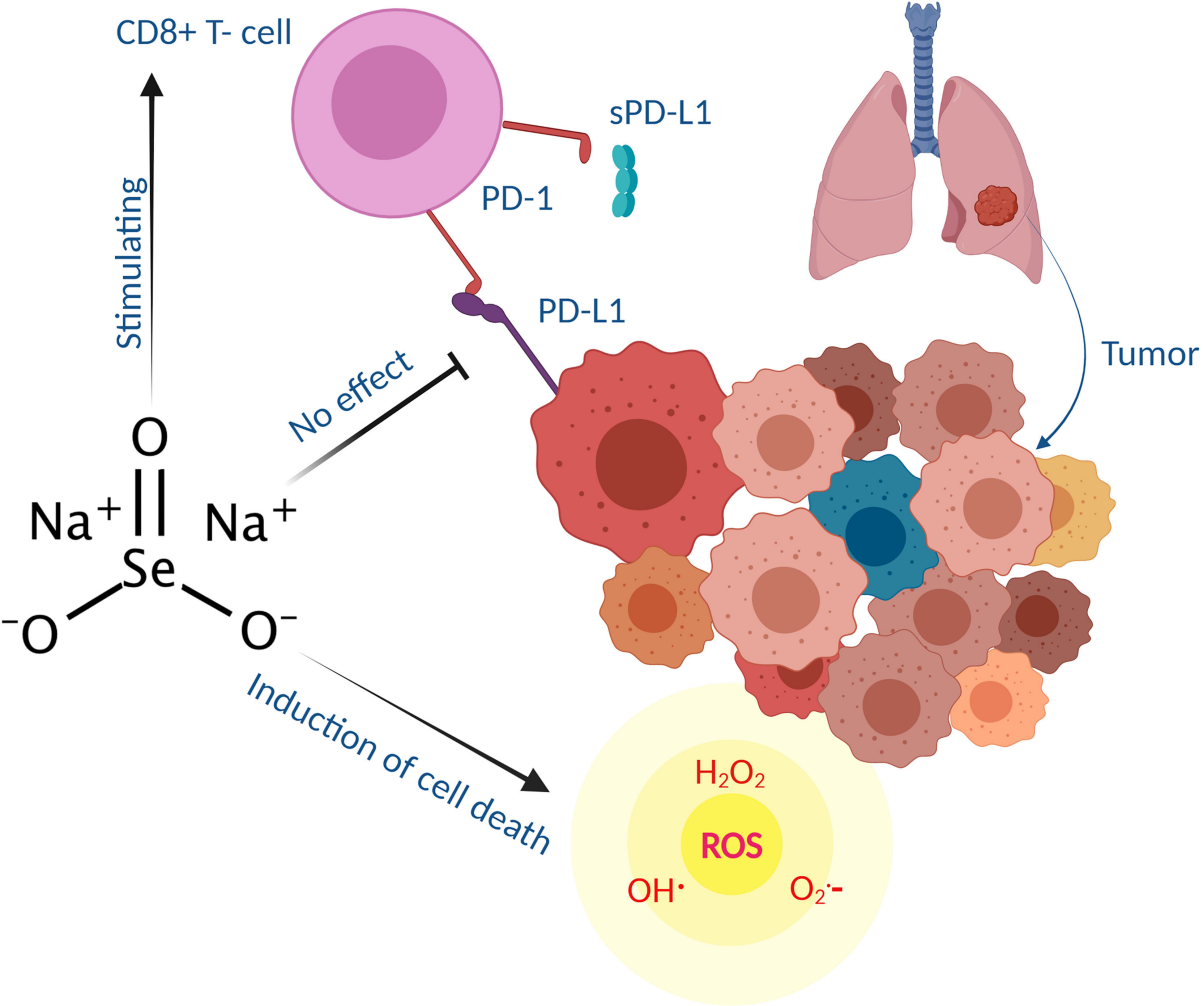
Schematic illustration showing that sodium selenite stimulates cytotoxic CD8 T -cells induce cell death through reactive oxygen species (ROS) generation in cancer patients (mostly lung adenocarcinoma) while having no effects on PD-L1 expression. Biologically, sPD-L1 inhibits the proliferation of T cells (particularly CD8+ subgroup) by PD-L1/PD-1 cross talk thus associated with immunosuppression (14).

The effect of selenotherapy on PD-L1 expression is controversial. Our study supports the view that selenotherapy does not on an aggregated data level substantially affect the PD-1/PD-L1 axis judged by sPD-L1 analysis (**Figure 2**). Moreover, no significant correlations were noted between the survival and sPD-L1 levels (before treatment, during the treatment period, or at the end of treatment) and changes of sPD-L1 levels. However, our data display a dynamic and individual change in sPD-L1 levels during selenotherapy as well as a high variation in survival, which does not rule out the possibility that individual patients may be benefited from combinational selenotherapy and anti-PD-L1 therapy. In the future, it would be of interest to study the serum levels of both sPD-1 and sPD-L1, as well as, PD-L1 immunohistochemistry to gain more vision into the effects of selenium on sPD-L1.

## Data Availability Statement

The original contributions presented in the study are included in the article/supplementary material. Further inquiries can be directed to the corresponding authors.

## Ethics Statement

The study was approved by the Ethical Committee of Stockholm and the Swedish Medical Products Agency (2006/429-31/3), registered in EU Clinical Trial Register (Eudra CT Number: 2006-004076-13). The patients/participants provided their written informed consent to participate in this study.

## Author Contributions

AR performed the analysis and wrote the manuscript. LM conducted the analysis. OB performed clinical data collection. MB supervised the project. JL reviewed the analysis and manuscript and supervised the project. All authors contributed to the article and approved the submitted version.

## Funding

This investigation was supported by grants from Cancerfonden, Cancer och Allergifonden, Radiumhemmets Forskningsfonder, and Sjöbergstiftelsen to MB. The funders had no role in study design, data collection, analysis, the decision to publish, or preparation of the manuscript.

## Conflict of Interest

MB is listed as an inventor in a patent application for *i.v.* use of inorganic selenium in cancer patients and holds shares in SELEQ OY, a company involved in the development of Se-based formulations for prevention and treatment.

The remaining authors declare that the research was conducted in the absence of any commercial or financial relationships that could be construed as a potential conflict of interest.

## Publisher’s Note

All claims expressed in this article are solely those of the authors and do not necessarily represent those of their affiliated organizations, or those of the publisher, the editors and the reviewers. Any product that may be evaluated in this article, or claim that may be made by its manufacturer, is not guaranteed or endorsed by the publisher.
